# Non-contiguous finished genome sequence and description of *Collinsella massiliensis* sp. nov.

**DOI:** 10.4056/sigs.5399696

**Published:** 2014-03-15

**Authors:** Roshan Padmanabhan, Gregory Dubourg, Jean-Christophe lagier, Thi-Thien Nguyen, Carine Couderc, Morgane Rossi-Tamisier, Aurelia Caputo, Didier Raoult, Pierre-Edouard Fournier

**Affiliations:** 1 Unité de Recherche sur les Maladies Infectieuses et Tropicales Emergentes, Institut Hospitalo-Universitaire Méditerranée-Infection, Faculté de médecine, Aix-Marseille Université, Marseille cedex 05, France; 2 King Fahd Medical Research Center, King Abdulaziz University, Jeddah, Saudi Arabia

**Keywords:** *Collinsella massiliensis*, genome, culturomics, taxono-genomics

## Abstract

*Collinsella massiliensis* strain GD3^T^ is the type strain of *Collinsella massiliensis* sp. nov., a new species within the genus *Collinsella*. This strain, whose genome is described here, was isolated from the fecal flora of a 53-year-old French Caucasoid woman who had been admitted to intensive care unit for Guillain-Barré syndrome. *Collinsella massiliensis* is a Gram-positive, obligate anaerobic, non motile and non sporulating bacillus. Here, we describe the features of this organism, together with the complete genome sequence and annotation. The genome is 2,319,586 bp long (1 chromosome, no plasmid), exhibits a G+C content of 65.8% and contains 2,003 protein-coding and 54 RNA genes, including 1 rRNA operon.

## Introduction

*Collinsella massiliensis* strain GD3^T^ (= CSUR P902 = DSM 26110) is the type strain of *C. massiliensis* sp. nov. This bacterial strain was isolated from the fecal flora of a 53-year-old French Caucasoid female admitted to the intensive care unit (ICU) in the Timone Hospital of Marseille, France, for Guillain-Barré syndrome. This study was part of a “culturomics” effort to cultivate all bacteria within human feces [1]. *C. massiliensis* is a Gram-positive, obligatly anaerobic, non-endospore forming, non-motile and rod shaped bacillus.

Thanks to the development of high throughput sequencers and the rapidly declining cost of genome sequencing, the number of sequenced bacterial genomes has reached almost 12,000 as of January 2^nd^, 2014, with an additional 18,000 sequencing projects ongoing [2]). In an effort to include genomic information among the genotypic criteria used for the taxonomic description of bacterial isolates, and not only rely on a combination of 16S rRNA gene phylogeny and nucleotide sequence similarity, G + C content and DNA–DNA hybridization [3-6]. We proposed a new strategy named taxono-genomics that we used to describe several new bacterial taxa [7-38].

In 1999, Kageyama *et al.* reclassified *Eubacterium aerofaciens* into a new genus named *Collinsella* [39] based on a 16S rRNA gene sequence divergence and the presence of a unique peptidoglycan type when compared to other members of the genus *Eubacterium*. In addition to the type species, *C. aerofaciens* [39], the genus *Collinsella* currently includes *C. intestinalis* [40], *C. stercoris* [40] and *C. tanakaei* [41]. All four species have been isolated from the human gastrointestinal tract.

In the present manuscript, we apply the taxono-genomics strategy to the description of *Collinsella massiliensis* sp. nov., and describe the complete genome sequencing and annotation of *Collinsella massiliensis* strain GD3^T^ (= CSUR P902 = DSM 26110). These characteristics support the circumscription of the *C. massiliensis* species.

## Classification and Features

A stool sample was collected from a 53-year-old female admitted to the intensive care unit of the Timone Hospital in Marseille, France, for Guillain-Barré syndrome. The patient gave a written informed consent for the study, which was approved by the Ethics Committee of the Institut Fédératif de Recherche 48, Faculty of Medicine, Marseille, France, under agreement number 09-022. She received antibiotics at the time of stool sample collection and the fecal specimen was preserved at -80°C immediately after collection. Strain GD3^T^ (Table 1) was first isolated in January 2012 after incubation for two weeks in an anaerobic blood culture bottle that also contained clarified and sterile sheep rumen. Then, the strain was sub-cultivated anaerobically at 37°C on 5% sheep blood-enriched Columbia agar (BioMerieux, Marcy l’Etoile, France). Several other new bacterial species were isolated from this stool specimen using various culture conditions.

**Table 1:** Classification and general features of *Collinsella massiliensis* strain GD3^T^ according to the MIGS recommendations [42].

**MIGS ID**	**Property**	**Term**	**Evidence code^a^**
	Current classification	Domain *Bacteria*	TAS [43]
		Phylum *Actinobacteria*	TAS [44]
		Class *Actinobacteria*	TAS [45]
		Order *Coriobacteriales*	TAS [45,46]
		Family *Coriobacteriaceae*	TAS [47]
		Genus *Collinsella*	TAS [39]
		Species *massiliensis*	IDA
		Type strain GD3^T^	IDA
	Gram stain	Positive	IDA
	Cell shape	Bacilli	IDA
	Motility	Non motile	IDA
	Sporulation	Non spore forming	IDA
	Temperature range	Mesophilic	IDA
	Optimum temperature	37°C	IDA
MIGS-6.3	Salinity	Unknown	NAS
MIGS-22	Oxygen requirement	Anaerobic	IDA
	Carbon source	Unknown	NAS
	Energy source	Unknown	NAS
MIGS-6	Habitat	Human gut	IDA
MIGS-15	Biotic relationship	Free living	IDA
MIGS-14	Pathogenicity Biosafety level Isolation	Unknown 2 Human feces	IDA
MIGS-4	Geographic location	France	IDA
MIGS-5	Sample collection time	January 2012	IDA
MIGS-4.1	Latitude	43.296482	IDA
MIGS-4.1	Longitude	5.36978	IDA
MIGS-4.3	Depth	Surface	IDA
MIGS-4.4	Altitude	0 m above sea level	IDA

Evidence codes - IDA: Inferred from Direct Assay; TAS: Traceable Author Statement (i.e., a direct report exists in the literature); NAS: Non-traceable Author Statement (i.e., not directly observed for the living, isolated sample, but based on a generally accepted property for the species, or anecdotal evidence). These evidence codes are from the Gene Ontology project [48]. If the evidence is IDA, then the property was directly observed for a live isolate by one of the authors or an expert mentioned in the acknowledgements.

**Figure 1:**
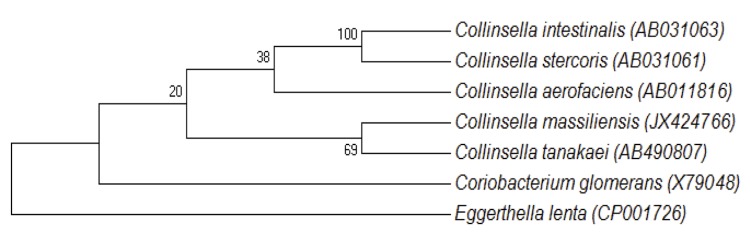
A consensus phylogenetic tree highlighting the position of *Collinsella massiliensis* strain GD3^T^ relative to other type strains within the genus *Collinsella*. GenBank accession numbers are indicated in parentheses. Sequences were aligned using CLUSTALW and phylogenetic inferences obtained using the neighbor-joining method within the MEGA software. Numbers at the nodes are percentages of bootstrap values obtained analysis from1,000 REPLICTES to generate a majority consensus tree. *Eggerthella lenta* was used as an outgroup.

When compared to sequences available in GenBank, the 16s rRNA sequence of *C. massiliensis* strain GD3^T^ (GenBank accession number JX424766) exhibited the highest sequence identity of 95.7% with *Collinsella tanakaei* (Figure 1). This value was lower than the threshold (98.7%) recommended by Stackebrandt and Ebers to delineate a new species without carrying out DNA-DNA hybridization [4], and was in the range of 16S rRNA identity values observed among the four *Collinsella* species with validly published names (92.2 between *C. intestinalis* and *C. aerofaciens* to 97.7% between *C. intestinalis* and *C. stercoris*) [49].

**Figure2:**
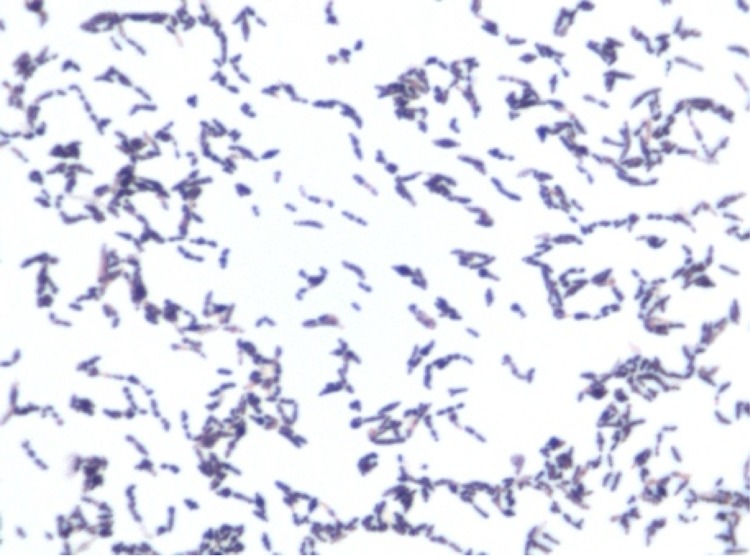
Gram staining of *Collinsella massiliensis* strain GD3^T^

**Figure 3:**
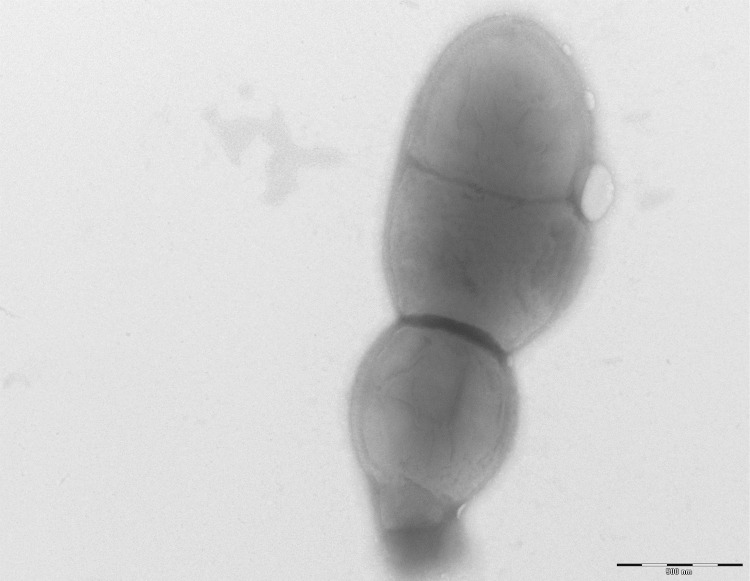
Transmission electron microscopy of *Collinsella massiliensis* strain GD3^T^, made using a Morgagni 268D (FEI Electron Optics, Hillsboro, OR, USA) at an operating voltage of 60 kV. The scale bar represents 500 µm.

Growth of the strain was tested in 5% sheep blood-enriched Columbia agar (BioMerieux) under anaerobic and microaerophilic conditions (GENbag anaer and GENbag microaer systems, respectively, BioMerieux), and in aerobic conditions, with or without 5% CO_2_. Growth was achieved only anaerobically. In addition, among the four different incubation temperatures tested (25, 30, 37, 45°C), no growth was observed at 25°C and 30°C but strain GD3^T^ grew at 37 and 45°C. The best growth was obtained at 37°C after 48 hours of incubation. Colonies were grey, translucent and 0.4 mm in diameter on blood-enriched Columbia agar. Gram staining showed Gram-positive rods unable to form spores (Figure 2). A motility test was negative. In electron microscopy, cells grown on agar had a mean diameter of 0.57µm, a mean length of 1.19µm (Figure 3) and were mostly grouped in short chains or small clumps.

Strain GD3^T^ showed neither catalase nor oxidase activities. Using an API ZYM strip (BioMerieux), positive reactions were observed for acid phosphatase, naphthol-AS-BI-phosphohydrolase, α-galactosidase, alkaline phosphatase, leucine arylamidase, α-glucosidase. Negative reactions were observed for cystin arylamidase, β-glucuronidase, nitrate reduction, urease, esterase (C4), esterase lipase (C8), lipase (C14), Trypsin, α-chemotrypsin, N-actetyl-β-glucosaminidase, α-mannosidase and α-fucosidase. Using an API Rapid ID 32A strip (BioMerieux), positive reactions were observed for α-galactosidase, α-glucosidase, α-fucosidase, leucine arylamidase, proline arylamidase, arginine dihydrolase, serine arylamidase and glycine arylamidase. Negative reactions were observed for histidin arylamidase, urease, phenylalanine arylamidase, tyrosin arylamidase, leucyl-glycyl arylamidase, alanine arylamidase, and arginine arylamidase. Using an API 50 CH strip (BioMerieux), positive reactions were obtained for D-sorbitol, D-saccharose, xylitol, D-arabitol and potassium-5-ketogluconate. Negative reactions were observed for the fermentation of glycerol, erythritol, D-arabinose, L-arabinose, D-ribose, D-xylose, L-xylose, D-adonitol, methyl-β-D-xylopranoside, D-galactose, D-glucose, D-fructose, D-mannose, L-sorbose, L-rhamnose, dulcitol, inositol, D-mannitol, methyl-αD-xylopranoside, methyl-αD-glucopranoside, N-acetylglucosamine, amygdalin, arbutin, esculin ferric citrate, salicin, D-cellobiose, D-maltose, D-lactose, D-mellibiose, D-trehalose, inulin, D-melezitose, D-raffinose, amidon, glycogen, gentiobiose, D-turanose, D-lyxose, D-tagatose, L-fucose, L-arabitol, potassium gluconate and potassium 2-ketogluconate. *Collinsella massiliensis* is susceptible to penicillin G, amoxicillin, amoxicillin-clavulanic acid, ceftriaxon, imipenem, metronidazole, vancomycin, rifampicin but resistant to erythromycin, gentamicin, ciprofloxacin and trimethoprim/sulfamethoxazole. By comparison with all other *Collinsella* species (Table 2), *C. massiliensis* differed in production of arginine arylamidase, leucine arylamidase, leucyl-glycyl arylamidase, and acidification of glucose, mannose, galactose, fructose and sorbitol.

**Table 2:** Differential characteristics of *Collinsella massiliensis* strain GD3^T^ with other strains

Properties	*C. massiliensis*	*C. intestinalis*	*C. aerofaciens*	*C. tanakei*	*C. stercoris*
Cell diameter (µm)	0.57	0.3-0.5	0.3-0.7	0.5-1.0	0.3-0.5
Oxygen requirement	anaerobic	anaerobic	anaerobic	anaerobic	anaerobic
Gram stain	+	+	+	+	+
Motility	-	-	na	-	-
Endospore formation	-	-	-	na	-
**Production of**					
Alkaline phosphatase	+	+	-	+	+
Acid phosphatase	+	+	-	+	+
Catalase	-	na	na	-	na
Oxidase	-	na	na	-	na
Nitrate reductase	-	na	na	-	na
Urease	-	na	-	-	na
α-galactosidase	+	-	+	-	-
β-galactosidase	+	-	+	-	+
β-glucuronidase	-	-	-	+	-
α -glucosidase	+	-	+	-	-
β-glucosidase	-	-	-	+	+
Esterase	-	na	-	-	na
Esterase lipase	-	-	-	-	-
Indole	-	na	na	-	na
N-acetyl-β-glucosaminidase	-	+	-	-	+
Alanine arylamidase	-	na	-	-	+
Arginine arylamidase	-	na	+	+	na
Cystin arylamidase	-	-	-	-	-
Glutamic acid decarboxylase	-	na	-	-	na
Glycine arylamidase	+	na	+	+	na
Leucine arylamidase	-	+	+	+	+
Leucyl-glycyl arylamidase	-	na	+	+	na
Proline arylamidase	+	-	+	+	-
Serine arylamidase	+	na	-	-	+
Tyrosin arylamidase	-	na	-	-	+
Valine arylamidase	-	-	+	-	-
**Utilization of**					
Glucose	-	+	+	+	+
Mannose	-	+	+	+	+
Galactose	-	+	+	na	+
Fructose	-	+	+	na	+
Maltose	-	-	+	+	+
Cellobiose	-	-	-	+	+
Lactose	-	-	+	+	+
L-arabinose	-	-	-	-	-
D-xylose	-	-	-	-	-
Rhamnose	-	-	na	-	-
Ribose	-	-	+	+	+
Raffinose	-	-	na	na	-
Glycogen	-	-	na	na	-
Aesculin	-	-	-	-	-
Mannitol	-	-	-	na	-
Sorbitol	+	-	-	-	-
Habitat	human gut	human gut	human gut	human gut	na

na: data not available

+/-: depending on tests used

**Figure 4:**
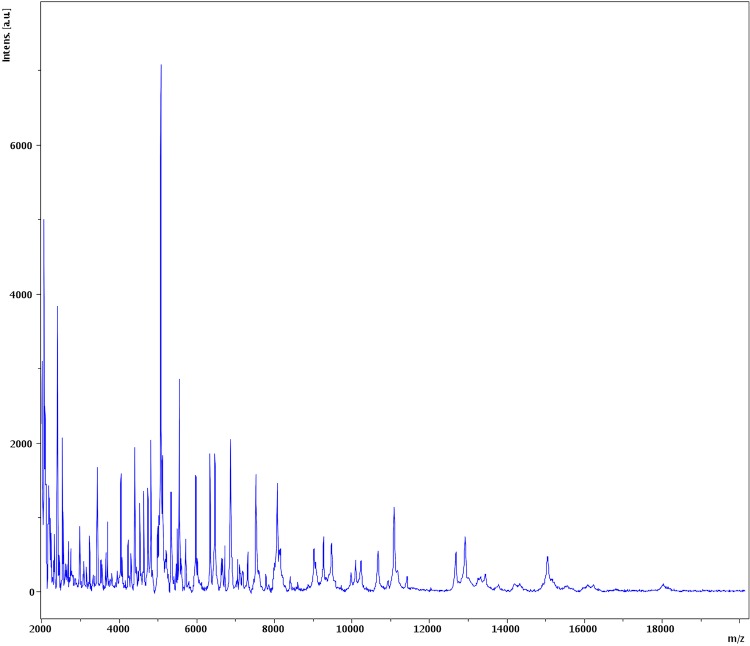
Reference mass spectrum from *Collinsella massiliensis* strain GD3^T^. Spectra from 12 individual colonies were compared and a reference spectrum was generated.

**Figure 5:**
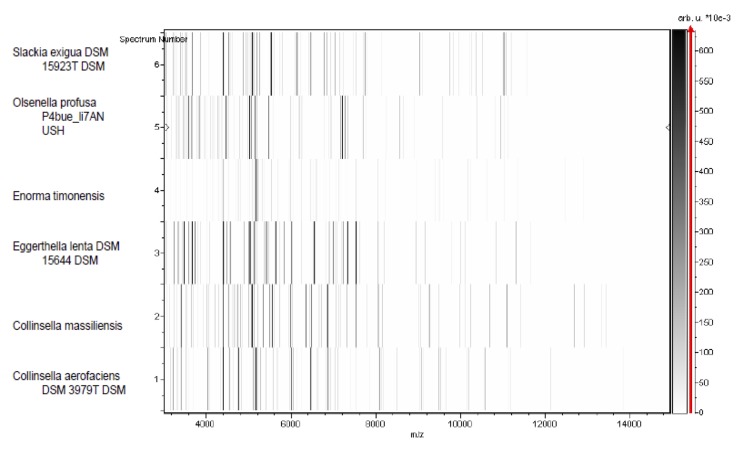
Gel view comparing *Collinsella massiliensis* strain GD3^T^ to other members of the family *Coriobacteriaceae*. The gel view displays the raw spectra of all loaded spectrum files arranged in a pseudo-gel-like look. The x-axis records the m/z value. The left y-axis displays the running spectrum number originating from subsequent spectra loading. The peak intensity is expressed by a Gray scale scheme code. The color bar and the right y-axis indicate the relation between the color a peak is displayed with and the peak intensity in arbitrary units. Displayed species are detailed in the left column.

Matrix-assisted laser-desorption/ionization time-of-flight (MALDI-TOF) MS protein analysis was peformed as previously described [50] using a Microflex spectrometer (Bruker Daltonics, Leipzig, Germany). The spectra from 12 distinct colonies from a culture agar plate were imported into the MALDI BioTyper software (version 2.0, Bruker) and analyzed by standard pattern matching (with default parameter settings) against the main spectra of 4,706 bacteria including 2 spectra from *Collinsella aerofaciens*, that were part of the reference data contained in the BioTyper database. The resulting score enabled the presumptive identification and discrimination of the tested isolate from those in the database according to the following rule: a score > 2 with a validated species enabled the identification at the species level; a score > 1.7 but < 2 enabled the identification at the genus level; and a score < 1.7 did not enable any identification. No significant score was obtained for strain GD3^T^, suggesting that the isolate was not a member of any known species. The reference mass spectrum of *Collinsella massiliensis* strain GD3^T^ and the gel view comparing this spectrum with other phylogenetically close species are presented in Figures 4 and 5, respectively.

## Genome sequencing information

### Genome project history

The organism was selected for sequencing on the basis of its phylogenetic position and 16S rRNA similarity to members of the genus *Colinsella*, and is part of a study of the human digestive flora aiming at isolating all bacterial species within human feces [1]. It was the fifth genome of a *Colinsella* species and the first genome of *C. massiliensis* sp. nov. The GenBank accession number is CAPI00000000 and consists of 15 scaffolds and 118 large contigs. Table 3 shows the project information and its association with MIGS version 2.0 compliance [42].

**Table 3:** Project information

**MIGS ID**	**Property**	**Term**
MIGS-31	Finishing quality	High-quality draft
MIGS-28	Libraries used	One 454 paired-end 5-kb library
MIGS-29	Sequencing platforms	454 GS FLX Titanium
MIGS-31.2	Fold coverage	92 ×
MIGS-30	Assemblers	Newbler version 2.5.3
MIGS-32	Gene calling method	Prodigal
	INSDC ID /GenBank ID	CAPI00000000
	BioProject ID	PRJEB541
	Genbank Date of Release	17/12/2012
	Project relevance	Study of the human gut microbiome

### Growth conditions and DNA isolation

*Collinsella massiliensis* strain GD3^T^ (= CSUR P902 = DSM 26110) was grown on 5% sheep blood-enriched Columbia agar (BioMerieux) at 37°C in anaerobic atmosphere. Bacteria grown on four Petri dishes were harvested and resuspended in 4x100µL of TE buffer. Then, 200µL of this suspension was diluted in 1ml TE buffer for lysis treatment that included a 30- minute incubation with 2.5 µg/µL lysozyme at 37°C, followed by an overnight incubation with 20 µg/µL proteinase K at 37°C. Extracted DNA was then purified using 3 successive phenol-chloroform extractions and ethanol precipitation at -20°C overnight. Following centrifugation, the DNA was resuspended in 52 µL TE buffer. The yield and concentration was measured by the Quant-it Picogreen kit (Invitrogen) on the Genios-Tecan fluorometer at 26.3 ng/µl.

### Genome sequencing and assembly

Five µg of DNA was mechanically fragmented on Covaris device (KBioScience-LGC Genomics, Teddington, UK) using miniTUBE-red. The DNA fragmentation was visualized through an Agilent 2100 BioAnalyzer on a DNA labchip 7500 with an optimal size of 1.9kb. A 5 kb paired-end library was constructed according to the 454 GS FLX Titanium paired-end protocol (Roche). Circularization and nebulization were performed and generated a pattern with an optimal at 567 bp. After PCR amplification through 17 cycles followed by double size selection, the single stranded paired-end library was quantified with the Quant-it Ribogreen kit (Invitrogen) on the Genios Tecan fluorometer at 505pg/µL. The library concentration equivalence was calculated as 8.17E+09 molecules/µL. The library was stored at -20°C until further use.

The paired-end library was clonally amplified with 0.5cpb and 1cbp in 4 SV-emPCR reactions with the GS Titanium SV emPCR Kit (Lib-L) v2 (Roche). The yields of the emPCR reactions were 9.35 and 14.76% respectively, in the range of 5 to 20% from the Roche procedure. The library was loaded on a GS Titanium PicoTiterPlate PTP Kit 70x75 and sequenced with the GS Titanium Sequencing Kit XLR70 (Roche). The run was performed overnight and then analyzed on the cluster through the gsRunBrowser and Newbler assembler (Roche). A total, of 672,867 passed filter wells were obtained and generated 214.2Mb with a length average of 301bp. These sequences were assembled using Newbler (Roche) with 90% identity and 40bp as overlap. The final assembly identified 15 scaffolds and 118 large contigs (>1500bp) generating a genome size of 2.32 Mb which corresponds to a coverage of 92x genome equivalent.

### Genome annotation

Open Reading Frames (ORFs) were predicted using Prodigal [51] with default parameters. However, when predicted ORFs spanned a sequencing gap region, they were excluded. The predicted bacterial protein sequences were searched against the GenBank [52] and Clusters of Orthologous Groups (COG) databases using BLASTP. The tRNAScan-SE [53] and RNAmmer [54] softwares were used to predict tRNAs and rRNAs, respectively. Signal peptides and numbers of transmembrane helices were predicted using SignalP [55] and TMHMM [56], respectively. Mobile genetic elements were predicted using PHAST [57] and RAST [58]. ORFans were identified if their BLASTP *E*-value was lower than 1e-03 for alignment length greater than 80 amino acids. If alignment lengths were smaller than 80 amino acids, we used an *E*-value of 1e-05. Such parameter thresholds have already been used in previous works to define ORFans. Artemis [59] and DNA Plotter [60] were used for data management and visualization of genomic features, respectively. Mauve alignment tool (version 2.3.1) was used for multiple genomic sequence alignment [61].

To estimate the mean level of nucleotide sequence similarity at the genome level between *C. massiliensis* and the other 4 members of the genus *Collinsella* (Table 6), we used the Average Genomic Identity Of gene Sequences (AGIOS) home-made software [7]. Briefly, this software combines the Proteinortho software [62] for detecting orthologous proteins between genomes compared two by two, then retrieves the corresponding genes and determines the mean percentage of nucleotide sequence identity among orthologous ORFs using the Needleman-Wunsch global alignment algorithm. *C. massiliensis* strain GD3^T^ was compared to *C. intestinalis* strain DSM 13280 (GenBank accession number ABHX00000000), *C. aerofaciens* strain ATCC 25986 (AAVN00000000), *C. stercoris* strain DSM 13279 (ABXJ00000000), *C. tanakaei* strain YIT 12063 (ADLS00000000), *Eggerthella lenta* strain DSM 2243 (CP001726) and *Coriobacterium glomerans* strain PW2 (CP0002628).

**Table 6:** Genomes used in the genomic comparison and their main characteristics

**Organism**	**GenBank id**	**Size (Mb)**	**GC%**	**Genes**
*Collinsella massiliensis* GD3	CAPI00000000	2.32	65.8	2,054
*Collinsella intestinalis* DSM 13280	ABXH00000000	1.8	62.5	1,846
*Collinsella aerofaciens* ATCC 25986	AAVN00000000	2.44	60.6	2,437
*Collinsella stercoris* DSM 13279	ABXJ00000000	2.4	63.2	2,585
*Collinsella tanakaei* YIT 12063	ADLS00000000	2.48	60.2	2,276
*Eggerthella lenta* DSM 2243	CP001726	3.63	64.2	3,184
*Coriobacterium glomerans* PW2	CP002628	2.12	60.4	1,858

## Genome properties

**Figure 6:**
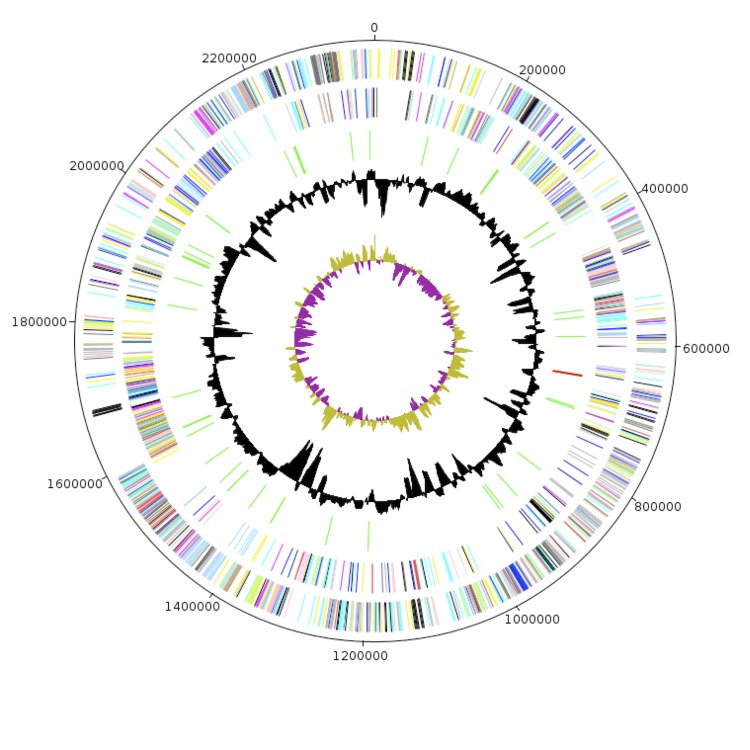
Graphical circular map of the *Collinsella massiliensis* strain GD3^T^ chromosome. From the outside in: open reading frames oriented in the forward (colored by COG categories) direction, open reading frames oriented in the reverse (colored by COG categories) direction, RNA operon (red), and tRNAs (green), GC content plot, and GC skew (purple: negative values, olive: positive values).

The genome of *C. massiliensis* strain GD3^T^ is 2,319,586 bp long (1 chromosome, no plasmid) with a 65.8% G+C content (Table 4 and Figure 6). Of the 2,057 predicted genes, 2,003 were protein-coding genes and 54 were RNAs (51 tRNA and 3 rRNA genes). A total of 1,503 genes (73.06%) were assigned a putative function. A total of 500 genes (24.30%) were annotated as hypothetical proteins. The properties and the statistics of the genome are summarized in Tables 4 and 5. The distribution of genes into COGs functional categories is presented in Table 5. A total of 165 genes were identified as ORFans (8.02%).

**Table 4:** Nucleotide content and gene count levels of the genome

**Attribute**	Value	% of total^a^
Genome size (bp)	2,319,586	
DNA G+C content (bp)	1,526,287	65.8
DNA coding region (bp)	1,997,199	86.10
Number of replicons	1	
Extrachromosomal elements	0	
Total genes	2,054	100
RNA genes	54	2.62
rRNA operons	1	
Protein-coding genes	2,003	97.37
Genes with function prediction	1,503	73.06
Genes assigned to COGs	1,370	66.60
Genes with peptide signals	40	1.94
Genes with transmembrane helices	471	22.89
CRISPR repeats	2	

^a^ The total is based on either the size of the genome in base pairs or the total number of protein-coding genes in the annotated genome.

**Table 5:** Number of genes associated with the 25 general COG functional categories

**Code**	**Value**	**% of total**^a^	**Description**
J	135	6.73	Translation
A	0	0	RNA processing and modification
K	113	5.64	Transcription
L	79	3.94	Replication, recombination and repair
B	0	0	Chromatin structure and dynamics
D	18	0.89	Cell cycle control, mitosis and meiosis
Y	0	0	Nuclear structure
V	53	2.64	Defense mechanisms
T	29	1.44	Signal transduction mechanisms
M	81	4.04	Cell wall/membrane biogenesis
N	2	0.09	Cell motility
Z	0	0	Cytoskeleton
W	0	0	Extracellular structures
U	10	0.49	Intracellular trafficking and secretion
O	39	1.94	Posttranslational modification, protein turnover, chaperones
C	69	3.44	Energy production and conversion
G	189	9.43	Carbohydrate transport and metabolism
E	123	6.14	Amino acid transport and metabolism
F	43	2.14	Nucleotide transport and metabolism
H	28	1.39	Coenzyme transport and metabolism
I	28	1.39	Lipid transport and metabolism
P	46	2.29	Inorganic ion transport and metabolism
Q	3	0.14	Secondary metabolites biosynthesis, transport and catabolism
R	165	8.23	General function prediction only
S	117	5.84	Function unknown
-	500	24.96	Not in COGs

^a^ The total is based on the total number of protein coding genes in the annotated genome.

## Genome comparison with other *Collinsella* genomes

**Figure 7:**
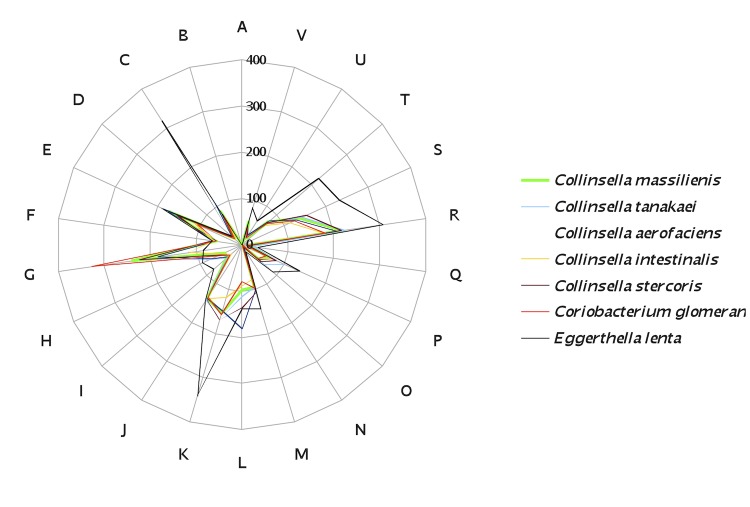
Distribution of functional classes of predicted genes of *C. massiliensis* sp. nov. strain GD3^T^ (green) and other members of the genus *Collinsella*, *Eggerthella lenta* and *Coriobacterium glomerans*.

The genome of *C. massiliensis* was compared with those of *C. intestinalis*, *C. aerofaciens*, *C. stercoris*, *C. tanakaei*, *Eggerthella lenta* and *Coriobacterium glomerans* (Table 6). The draft genome of *C. massiliensis* is larger than that of *C. intestinalis* and *C. glomerans* (2.32, 1.8 and 2.12 Mb, respectively) but smaller than all other other studied genomes (Table 6). In contrast, it exhibits a higher G+C content than all other genomes (Table 6). The distribution of genes into COG categories in the genomes from all 5 compared *Collinsella* species and *Coriobacterium glomerans* was similar but different from *Eggerthella lenta* (Figure 7). In addition, *C. massiliensis* shared 867, 947, 953, 1,029, 751 and 841 orthologous genes with *C. intestinalis, C. aerofaciens*, *C. stercoris*, *C. tanakaei, Eggerthella lenta* and *Coriobacterium glomerans*, respectively. Among compared *Collinsella* genomes except *C. massiliensis*, AGIOS values ranged from 74.19 between *C. aerofaciens* and *C. tanakaei* to 81.80% between *C. intestinalis* and *C. stercoris*. When *C. massiliensis* was compared to other *Collinsella* species, AGIOS values ranged from 74.37 with *C. tanakaei* to 76.52% with *C. stercoris* (Table 7). In addition, *C. massiliensis* exhibited AGIOS values of 71.24 and 73.73% with *Eggerthella lenta* and *Coriobacterium glomerans*, respectively (Table 7).

**Table 7:** Numbers of orthologous protein-coding genes shared among genomes

	CM	CA	CI	CS	CT	EL	CG
CM	**2,003**	74.61	75.95	76.51	74.37	71.24	73.73
CA	947	**2,367**	74.91	75.05	74.19	68.55	71.63
CI	867	945	**1,784**	81.80	75.67	69.28	72.42
CS	953	999	1,110	**2,529**	75.70	69.88	72.53
CT	1,029	1,082	1,108	1,151	**2,212**	68.46	71.50
EL	751	764	740	777	856	**3,070**	68.52
CG	841	841	818	861	898	639	**1,768**

Orthlogues, Lower triangle, AGIOS values upper triangle; number of proteins per genome (bold numbers). CM = *C. massiliensis*, CA = *C. aerofaciens*, CI = *C. intestinalis*, CS = *C. stercoris*, CT = *C. tanakaei*, EL = *Eggerthella lenta*, CG = *Coriobacterium glomerans.*

## Conclusion

On the basis of phenotypic, phylogenetic and genomic analyses (taxono-genomics), we formally propose the creation of *Collinsella massiliensis* sp. nov. That contains strain GD3^T^ as type strain. The strain was isolated from the fecal flora of a 53-year-old woman hospitalized in ICU in Marseille, France, due to a Guillain-Barré syndrome.

### Description of *Collinsella massiliensis* strain sp. nov.

*Collinsella massiliensis* (mas.si.li.en′sis. L. masc. adj. massiliensis of Massilia, the Roman name of Marseille, France, where type strain GD3^T^ was isolated).

Colonies are grey, translucent and 0.4 mm in diameter on blood-enriched Columbia agar. Cells are rod shaped with a mean diameter and length of 0.57 and 1.19 µm, respectively. Optimal growth is achieved anaerobically only. Growth occurs at 37 and 45°C, with optimal growth observed at 37°C.

Cells stain Gram-positive, are non-endospore forming and are non-motile. Cells are negative for catalase and oxidase. Positive reactions are observed for acid phosphatase, alkaline phosphatise, naphthol-AS-BI-phosphohydrolase, α -galactosidase, α-galactosidase, α-glucosidase, α-fucosidase, leucine arylamidase, proline arylamidase, arginine dihydrolase, serine arylamidase, glycine arylamidase and acidification of D-sorbitol, D-saccharose, xylitol, D-arabitol and potassium-5-ketogluconate. Negative reactions are observed for leucine arylamidase, valine arylamidase, cystin arylamidase, β-glucuronidase, nitrate reduction, urease, esterase (C4), esterase lipase (C8), lipase (C14), Trypsin, α-chemotrypsin, N-actetyl-β-glucosaminidase, α-mannosidase, α-fucosidase, histidin arylamidase, urease, phenylalanine arylamidase, tyrosin arylamidase, leucyl-glycyl arylamidase, alanine arylamidase, arginine arylamidase and fermentation of glycerol, erythritol, D-arabinose, L-arabinose, D-ribose, D-xylose, L-xylose, D-adonitol, methyl-β-D-xylopranoside, D-galactose, D-glucose, D-fructose, D-mannose, L-sorbose, L-rhamnose, dulcitol, inositol, D-mannitol, methyl-αD-xylopranoside, methyl-αD-glucopranoside, N-acetylglucosamine, amygdalin, arbutin, aesculin ferric citrate, salicin, D-cellobiose, D-maltose, D-lactose, D-mellibiose, D-trehalose, inulin, D-melezitose, D-raffinose, amidon, glycogen, gentiobiose, D-turanose, D-lyxose, D-tagatose, L-fucose, L-arabitol, potassium gluconate and potassium 2-ketogluconate. Positive reactions were recorded for acid phosphatase, naphthol-AS-BI-phosphohydrolase, α-galactosidase, alkaline phosphatase, leucine arylamidase, valine arylamidase, a-glucosidatse and β glucosidasee. Cells are susceptible to penicillin G, amoxicillin, amoxicillin-clavulanic acid, ceftriaxone, imipenem, metronidazole, vancomycin, rifampicin but resistant to erythromycin, gentamicin, ciprofloxacin and trimethoprim/sulfamethoxazole.

The 16S rRNA and genome sequences are deposited in GenBank and EMBL under accession numbers JX424766 and CAPI00000000, respectively. The G+C content of the genome is 65.8%. The habitat of the microorganism is the human digestive tract. The type strain GD3^T^ (= CSUR P902 = DSM 26110) was isolated from the fecal flora of a French Caucasoid woman who suffered from Guillain-Barré syndrome. This strain was isolated in Marseille, France.
